# Epigenetic Insights into Tuberous Sclerosis Complex, Von Hippel–Lindau Syndrome, and Ataxia–Telangiectasia

**DOI:** 10.3390/epigenomes9020020

**Published:** 2025-06-09

**Authors:** Gavriel Hadjigavriel, Christina Stylianides, Evangelos Axarloglou, Maria Eleni Manthou, Efstratios Vakirlis, Paschalis Theotokis, Soultana Meditskou, Iasonas Dermitzakis

**Affiliations:** 1 Department of Histology-Embryology, School of Medicine, Aristotle University of Thessaloniki, 54124 Thessaloniki, Greece; gavrchat@auth.gr (G.H.); christyl@auth.gr (C.S.); evanaxar@auth.gr (E.A.); mmanthou@auth.gr (M.E.M.); ptheotokis@auth.gr (P.T.); sefthym@auth.gr (S.M.); 2 First Department of Dermatology and Venereology, School of Medicine, Aristotle University of Thessaloniki, 54643 Thessaloniki, Greece; svakirlis@auth.gr

**Keywords:** neurocutaneous syndromes, epigenetics, tuberous sclerosis complex, von Hippel–Lindau syndrome, ataxia–telangiectasia, DNA methylation, histone modifications, non-coding RNAs

## Abstract

Neurocutaneous syndromes represent a clinically and genetically heterogeneous group of disorders, with tuberous sclerosis complex (TSC), von Hippel–Lindau syndrome (VHL), and ataxia–telangiectasia (A-T) exemplifying some of the most complex entities within this category. These syndromes have traditionally been considered monogenic disorders, caused by germline mutations in tumor suppressor or regulatory genes. However, they exhibit a striking degree of phenotypic variability and divergent clinical trajectories that cannot be fully explained by their underlying genetic alterations alone. Increasingly, epigenetic regulatory mechanisms, such as DNA methylation, histone modifications, chromatin remodeling, and non-coding RNA (ncRNA) activity, are recognized as key modulators of gene expression, cellular differentiation, and tissue-specific function. Disruption of these mechanisms has been implicated in disease pathogenesis, tumorigenesis, and neurodegeneration associated with TSC, VHL, and A-T. Aberrant epigenetic profiles may underlie the observed variability in clinical outcomes, even among individuals with identical mutations. This review consolidates current evidence on the epigenetic landscape of these syndromes, elucidating how these modifications may influence disease behavior and contribute to incomplete genotype–phenotype correlations. By integrating epigenetic insights with known molecular pathways, a more nuanced understanding of disease biology emerges, with potential implications for diagnostic stratification, prognostic assessment, and therapeutic innovation.

## 1. Introduction

Phakomatoses, also known as neurocutaneous syndromes, represent a diverse group of inherited disorders characterized by the involvement of structures derived from the embryonic ectoderm, notably the central nervous system and skin, but also the mesoderm, particularly the vascular system [1,2,3]. Neurocutaneous syndromes are paramount in clinical medicine due to their complex multisystem involvement, genetic underpinnings, and implications for patient management [4,5]. Neurofibromatosis type 1 (NF1) and Neurofibromatosis type 2 (NF2), tuberous sclerosis complex (TSC), von Hippel–Lindau disease (VHL), Sturge–Weber syndrome (SWS), and ataxia–telangiectasia (A-T) exemplify this group. Classically, phakomatoses have been viewed as monogenic disorders, typically resulting from germline mutations. Most follow an autosomal dominant inheritance pattern, except for Sturge–Weber syndrome, caused by a somatic mutation, and A-T, which is autosomal recessive [6,7]. Despite their monogenic nature, phakomatoses exhibit marked heterogeneity and multisystem involvement [8,9,10]. Different genetic causes often converge to produce similar neurocutaneous manifestations. A hallmark feature is their profound phenotypic variability, even among individuals carrying identical mutations. Factors such as mosaicism, variable expressivity, incomplete penetrance, and environmental influences can modulate disease severity, resulting in a broad clinical spectrum ranging from isolated skin lesions to life-threatening malignancies and neurological deficits [11]. This phenotypic diversity complicates early diagnosis, prognosis, and therapeutic planning, making individualized, multidisciplinary approaches essential.

Although both the development and pathological processes of ectoderm-derived tissues are primarily governed by genetic and molecular pathways, accumulating evidence suggests that epigenetic mechanisms also contribute significantly to the regulation of these processes [12,13,14,15,16,17]. Such mechanisms, including DNA methylation, histone modifications, and non-coding RNAs (ncRNAs), have been implicated in several ectodermal disorders, influencing gene expression without altering the DNA sequence and tissue-specific phenotypes. In parallel, epigenetic alterations have also been associated with aging, reinforcing their relevance in tissue degeneration and long-term disease progression [18]. Given this expanding understanding, it becomes increasingly pertinent to examine the role of epigenetic dysregulation in neurocutaneous syndromes such as TSC, VHL and A-T. Although NF1 and NF2 have been extensively characterized, with their pathogenesis primarily attributed to well-defined genetic mutations and increasingly recognized epigenetic mechanisms, other phakomatoses such as TSC, VHL, and A-T remain comparatively underexplored [19,20,21]. These syndromes merit intensified investigation not only because of their distinctive neurocutaneous manifestations but also due to the complex and dynamic interplay they exhibit between genetic alterations and epigenetic dysregulation [22]. Emerging evidence implicates aberrant DNA methylation, histone modifications, and dysregulation of ncRNAs as critical modulators of disease expression and progression.

Given the limited mechanistic insight currently available, this review examines the role of epigenetic regulation in the pathogenesis, prognosis, and management of TSC, VHL, and A-T. We explore how epigenetic modifications, including DNA methylation, histone remodeling, and ncRNA activity, contribute to disease expression and phenotypic variability and how these alterations correlate with clinical trajectories and therapeutic responses. This work synthesizes emerging evidence and outlines future research priorities to advance the understanding of these multifaceted syndromes.

## 2. Mapping the Epigenetic Architecture of TSC

TSC is a genetic disorder primarily caused by mutations in the TSC1 or TSC2 genes, leading to the dysregulation of the mechanistic target of rapamycin (mTOR) signaling pathway. However, emerging evidence suggests that epigenetic factors, including DNA methylation, histone modifications, and alterations in ncRNAs such as microRNAs (miRNAs), play a critical role in TSC. These epigenetic changes can affect key cellular functions, including proliferation, differentiation, and apoptosis, thereby contributing to TSC development. The complex interplay between genetic mutations and epigenetic modifications not only deepens our understanding of TSC’s varied clinical presentation but also holds the potential for developing more targeted and personalized treatment strategies for affected individuals.

### 2.1. Overview of TSC

TSC is an autosomal dominant, multisystem disorder characterized by cellular and tissue dysplasia in several organs. With the advent of genetic and molecular techniques, mutations in the *TSC1* or *TSC2* genes, which encode hamartin and tuberin, respectively, were discovered to be responsible for mTOR overactivation, which is the underlying mechanism of pathogenesis [23,24,25,26]. This dysregulation particularly affects the brain and skin, causing epilepsy, neurodevelopmental issues and hypopigmentation, and is linked to elevated vascular endothelial growth factor-D (VEGF-D) levels in conditions like lymphangioleiomyomatosis (LAM) and angiomyolipomas (AMLs) [27,28,29]. The gene mutation may be inherited or may occur spontaneously. Most cases present sporadically, with no known family history, but approximately 1 in 3 patients inherit a defective *TSC1* or *TSC2* gene [30]. The incidence of TSC has been estimated to occur in 1/6000–10,000 newborns annually, and therefore, it is categorized as a rare disease. It affects approximately 2 million people globally. The prevalence in Europe is approximately 11,500–25,000. There is no sex or ethnicity predilection [23]. 

TSC is a highly heterogeneous clinical entity with variable presentations and severity of disease. The brain, heart, skin, eyes, kidneys, and lungs are commonly involved in this syndrome, with neurologic symptoms comprising a significant source of morbidity and mortality. The hallmark of TSC is the development of hamartomas and tumors within various organ systems [22,31,32]. Even though there are no pathognomonic signs for TSC, various clinical symptoms are commonly seen as part of the syndrome, which raises suspicion about the diagnosis. Common manifestations include cortical tubers, subependymal nodules (SENs), subependymal giant cell astrocytomas (SEGAs), seizures, cardiac rhabdomyoma, renal AMLs, retinal hamartomas, pulmonary LAM, facial angiofibroma, ash-leaf spots, shagreen patches, intellectual disability, and autism spectrum disorder [23,25,33,34]. Heinrich Vogt’s 1908 clinical triad of epilepsy, intellectual disability, and adenoma sebaceous, now termed facial angiofibroma, was arguably the first crude diagnostic criteria for TSC. These criteria were revised in 2021.

Definite TSC is defined as the presence of either 2 major features or 1 major feature and 2 minor features. Possible TSC is defined as a single major feature or 2 or more minor features. In 2021, the TSC Clinical Consensus group concluded that the presence of a pathogenic variant in the *TSC1* or *TSC2* gene is sufficient for a diagnosis of TSC, regardless of clinical findings. SEGAs, AMLs, and LAM often arise from a second-hit mutation, though it is not always needed for cortical tubers. Also, about two-thirds of TSC cases are de novo, mostly involving TSC2. In the era of emerging preventive treatments and precision medicine, establishing a genetic diagnosis can help facilitate participation in clinical trials and access to new treatments [35]. In this perspective, the mTOR pathway and TSC genes are thought to be affected by epigenetic modifications, and growing evidence suggests that these alterations may contribute to the regulation of gene expression and the diverse clinical features observed in tuberous sclerosis complex [22].

### 2.2. The Contribution of Epigenetic Dysregulation to TSC Pathogenesis

#### 2.2.1. DNA Methylation

Methylation of the *TSC2* promoter was shown to silence the tuberin expression of *TSC2* in TSC-associated AMLs [36]. Smooth muscle-like cells were isolated from an AML. *TSC2* promoter methylation was analyzed using methylation-specific PCR and chromatin immunoprecipitation, while protein expression and cell proliferation were evaluated using complementary assays. These methylated cells, referred to as *TSC2*-/meth, exhibited markers such as HMB45 and CD44v6 and showed phosphorylation of S6, indicating activation of the mTOR pathway. Chromatin-remodeling agents like trichostatin A and 5-azacytidine reversed the methylation, restored tuberin expression, and normalized cell growth. Additionally, inhibition of EGFR or treatment with rapamycin effectively suppressed the proliferation of these cells. Lastly, the findings emphasized that *TSC2* promoter methylation might cause a complete loss of tuberin in TSC2 cells, suggesting that epigenetic defects in smooth muscle cells can cause the development of AMLs.

In addition to promoter methylation, broader DNA methylation alterations in SEGA tumors contribute to tumor heterogeneity. The role of DNA methylation in the development of SEGAs was investigated in TSC patients [37]. DNA was extracted from 42 formalin-fixed paraffin-embedded (FFPE) SEGA tumor specimens and eight periventricular control brain tissues. Genome-wide DNA methylation profiling was performed using the Illumina Infinium HumanMethylation450 BeadChip array. It was shown that methylation changes in SEGA were independent of *TSC1* or *TSC2* mutation status, and there was no evidence of promoter hypermethylation of *TSC1* or *TSC* silencing. Furthermore, DNA methylation alterations were not directly associated with mTOR pathway activation. Unsupervised clustering recognized two major SEGA subgroups (SEGA1 and SEGA2), with SEGA2 further subdividing into SEGA2a and SEGA2b. Although DNA methylation did not directly correlate with gene expression in the adaptive immune response or MAPK pathways, one subgroup showed increased T cell marker CD3 expression. This indicates that epigenetic changes related to immune function may modulate the tumor microenvironment and influence disease progression, thereby contributing to SEGA heterogeneity and potential therapeutic significance.

Epigenetic changes are also observed in the inflammatory aspect of TSC [38]. Specifically, hypomethylation in the interleukin-1b (IL-1β) promoter within tubers and surrounding regions plays a role in developing seizures in TSC. TSC is characterized by overexpression of various inflammation-related genes. Among the pro-inflammatory cytokines, IL-1β contributes significantly to both the onset of epilepsy and the persistence of seizures. In this context, the epigenetic regulation of neuroinflammation in TSC was investigated by analyzing TSC patients’ brain tissues and matching them with autopsy controls. Standard immunohistochemistry methods, such as antigen retrieval, antibody incubation, and confocal microscopy, were used to assess tissue morphology. In parallel, bisulfite modification, PCR amplification, cloning, and sequencing were performed to analyze methylation patterns within the IL-1β promoter. Significant hypomethylation of non-CpG cytosines in the IL-1β promoter in TSC brain tissues was observed, particularly in tubers and peritubular regions, which correlated with increased *IL-1β* gene expression. This hypomethylation is a characteristic feature of TSC neuropathology, such as epileptogenesis and maintenance of seizures, thus linking DNA methylation changes to the disease’s inflammatory pathogenesis.

Although the role of DNA methylation in TSC pathogenesis has been highlighted above, other research has found no such association. An extensive analysis of 111 TSC-associated tissues was conducted to explore, among other mechanisms, the role of promoter methylation in *TSC1*/*TSC2* inactivation [39]. Genetic and epigenetic data were integrated using whole-exome sequencing (WES), single nucleotide polymorphism (SNP) arrays, RNA sequencing (RNAseq), and HumanMethylation450 (HM450) arrays. Targeted deep sequencing was employed to detect low-frequency somatic mutations and intragenic deletions. No evidence of *TSC1* or *TSC2* promoter methylation was shown across the 63 samples analyzed for methylation, indicating that this epigenetic mechanism is unlikely to contribute significantly to gene inactivation in TSC. Finally, it was highlighted that mTOR activation in *TSC* is predominantly mutation-driven rather than epigenetically regulated via promoter methylation, reinforcing the primacy of genetic alterations in TSC pathogenesis.

#### 2.2.2. Histone Modifications

Building upon the role of epigenetic regulation in TSC, a recent study highlighted the contribution of histone modifications, particularly histone acetylation [40]. The contribution of chromatin remodeling, specifically the role of histone modifications, was investigated in TSC neurological deficits using a *Tsc2* haploinsufficient mouse model (*Tsc2*^+/−^). Histone acetylation levels were measured using immunohistochemistry, while synaptic plasticity was assessed through electrophysiological recordings and behavioral assays evaluated seizure thresholds. The effects of class I/II histone deacetylase inhibitors (HDACs) were tested. A reduction in histone H3 acetylation within the hippocampus of *Tsc2*^+/−^ mice was observed, which was associated with impaired synaptic plasticity and a new seizure phenotype. Interestingly, these effects were observed without significant changes in mTORC1 signaling, indicating that the neurophysiological improvements were mediated through mTOR-independent epigenetic mechanisms. To determine whether these epigenetic alterations were responsible for the observed neurological dysfunctions, treatment with inhibitors of class I/II histone deacetylases (HDACs) was administered. HDACs restored histone acetylation in *Tsc2*^+/−^ mice, improved synaptic function, and normalized seizure phenotype in line with wild-type mice. Overall, the results proposed a previously unrecognized role of chromatin modification in the pathophysiology of TSC-related neurodevelopmental and seizure phenotypes, highlighting the potential of HDACs in normalizing the impaired synaptic function and seizure susceptibility in *Tsc2*^+/−^ mice.

#### 2.2.3. Non-Coding RNAs

Emerging research has revealed that microRNAs contribute to the development of TSC by influencing gene regulation, mTOR signaling, and cellular differentiation processes. With regard to TSC-related epilepsy, the elevated expression of miRNAs in epileptogenic cortical tubers emphasizes the link between miRNA dysregulation and epileptogenesis. Brain tissue samples were taken from epileptogenic cortical tubers of five TSC patients, and healthy nearby brain tissue served as controls [41]. MiRNA profiling was performed, revealing four significantly overexpressed microRNAs: miR-23a, miR-34a, miR-34b, and miR-532-5p. To assess how these microRNAs affect their target proteins, quantitative proteomic profiling was performed using chromatography coupled with tandem mass spectrometry. The proportion of downregulated proteins among the predicted targets was significantly higher than in the overall proteome. This indicated an enrichment of proteins involved in synaptic signal transmission. Interestingly, the downregulated targets include *TSC1* and other epilepsy-related genes, with miR-23a and miR-34a directly suppressing hamartin expression. This links miRNA activity to synaptic dysfunction and the development of epilepsy in TSC. Furthermore, the study detected lower levels of hamartin in epileptogenic tubers and confirmed that miR-23a and miR-34a directly target the 3 ‘untranslated region (3 UTR) of the *TSC1*. Therefore, it was underscored that the epigenetic dysregulation of miRNAs contributes to the pathogenesis of TSC by affecting epileptogenic pathways and increasing seizure susceptibility in cortical tubers.

**Figure 1:**
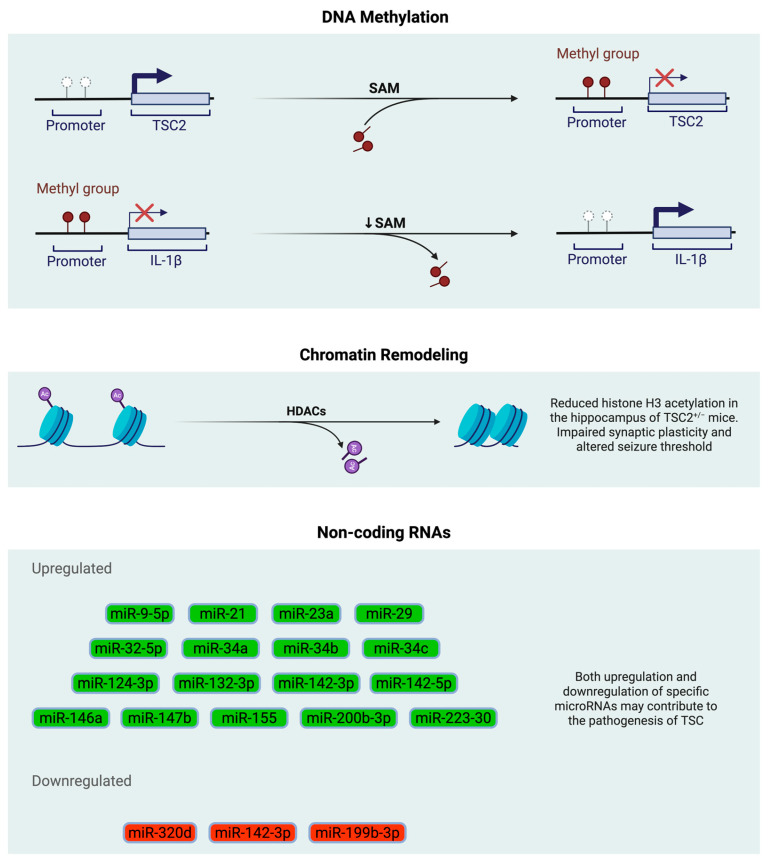
Epigenetic regulation of pathogenesis in tuberous sclerosis complex. DNA: deoxyribonucleic acid, HDAC: histone deacetylase, IL-1β: interleukin-1b, miRNA: microRNA, RNA: ribonucleic acid, SAM: s-adenosylmethionine, TSC2^+/−^: heterozygous mutations in the TSC2 gene, TSC: tuberous sclerosis complex. Created with BioRender.com.

Complementing this, an investigation of the relationship between miRNA expression and seizure onset in TSC patients revealed certain miRNAs related to cortical tubers’ metabolic and epileptogenic characteristics [42]. Brain tissue specimens were collected from TSC patients undergoing surgery for epilepsy. Both seizure and non-seizure onset tubers were analyzed using miRNA expression profiling. The epileptogenic status of each tuber was established using intracranial electrocorticography, while tryptophan metabolism was assessed with α-methyltryptophan positron emission tomography (AMT PET) imaging. Five specific miRNAS, namely miR-142-3p, miR-142-5p, miR-223-3p, miR-200b-3p and miR-32-5p, were found to distinguish among three tuber categories: non-onset/AMT-cold (NC), onset/AMT-cold (OC), and onset/AMT-hot (OH). These microRNAs were significantly upregulated in OH tubers and their expression correlated most strongly with AMT uptake, suggesting a link to metabolic and epileptogenic activity. Conversely, tubers without seizure onset and low AMT uptake or with seizure onset but reduced AMT uptake showed markedly lower levels of these miRNAs. Additionally, these miRNAs target genes that were confirmed to be involved in synaptic signaling and epilepsy risks, such as *SLC12A5*, *SYT1*, *GRIN2A*, *GRIN2B*, *KCNB1*, *SCN2A*, *TSC1* and *MEF2C*. Notably, a specific association between miR-32-5p and the *SLC12A5* gene was found. This points out a connection between miRNA alterations and the metabolic and epileptogenic features of TSC tubers, offering potential insights into the molecular mechanisms driving epilepsy in TSC patients (Figure 1).

Furthermore, the role of epigenetic regulation in TSC-associated SEGAs was investigated through miRNAs [43]. The expression of matrix metalloproteinases (MMPs), tissue inhibitors of metalloproteinases (TIMPs), and miR-320d were analyzed in SEGA tissues and compared to control brain tissues. This analysis was performed using RNA sequencing, real-time quantitative PCR (qPCR), and immunohistochemistry to assess transcript and protein levels. A significant overexpression of MMPs and TIMPs was revealed in SEGA samples. This indicates a dysregulated MMP/TIMP proteolytic system associated with extracellular matrix remodeling and tumorigenesis. In parallel, miR-320d was found to be downregulated. Functional assays in human fetal astrocytes showed that restoring miR-320d led to a reduction in MMP2 expression, suggesting a direct regulatory role of this miRNA in modulating MMP activity. Consistent with this, the importance of microRNA-mediated regulation of MMPs and TIMPs was further supported by studies in TSC-associated tubers [44]. In this context, the expression and localization of key MMPs and TIMPs in brain tissue from TSC patients were investigated using RT-PCR and immunohistochemistry. It was determined whether anti-inflammatory microRNAs, specifically miR146a and miR147b, can regulate MMP and TIMP expression in IL-1β-stimulated astroglial cultures derived from TSC tubers. The outcomes demonstrated an MMP/TIMP imbalance, characterized by a decrease in mRNA expression of TIMP2, TIMP3, and TIMP4 and an increase in MMP3 after IL-1β stimulation. Importantly, treatment with miR-146a and miR-147b in vitro reversed these changes in tuber-derived cell cultures. Therefore, these anti-inflammatory miRNAs could restore blood–brain barrier function and extracellular matrix balance, which are implicated in the pathogenesis of TSC-associated epilepsy.

In order to further discover how miRNAs epigenetically contribute to TSC pathogenesis, miRNA and mRNA expression patterns in TSC-related AML were analyzed [45]. RNA sequencing and qPCR were also employed to identify and validate miRNAs, and luciferase assays were used to confirm the direct suppression of *BCL2L11*, an apoptotic activator. The results showed 15 differentially expressed miRNAs and over 2600 dysregulated mRNAs, among which miR-9-5p, miR-124-3p, and miR-132-3p emerged as key regulators. In addition, in *Tsc2*^−/−^ cells, overexpression of these miRNAs enhanced proliferation and inhibited apoptosis, mirroring the effects of *BCL2L11* downregulation. Interestingly, restoring *BCL2L11* reversed these effects, confirming its function as a tumor suppressor epigenetically silenced by miRNA activity. These findings reveal a novel post-transcriptional regulatory network in TSC-AML, in which miRNA-mediated suppression of apoptosis-related genes like *BCL2L11* leads to tumor growth.

The role of inflammation-related miRNAs in TSC-associated brain lesions was further investigated [46,47]. Samples from cortical tubers and SEGA lesions were analyzed using quantitative real-time PCR to assess miRNA expression. In addition, human astrocyte and SEGA-derived cell cultures were employed with interleukin-1β (IL-1β) to investigate its impact on miRNA regulation. The results showed elevated miR21, miR146a and miR155 levels in TSC tubers compared to healthy brain tissue, particularly in dysmorphic neurons, giant cells and reactive astrocytes. Stimulation with IL-1b demonstrated increased expression of these intracellular microRNAs and extracellular expression of miR146a. Functionally, miR155 showed pro-inflammatory effects, while miR146a showed anti-inflammatory effects. The functional role of miRNAs produced by astrocytes in response to IL-1β-induced inflammation was also investigated in epilepsy. Sequencing of miRNA and mRNA in IL-1β-stimulated human fetal astrocytes revealed significant upregulation of miR-146a and miR-147b. Overexpression of these miRNAs using mimics led to a 40–50% reduction in cyclooxygenase-2 (COX-2) and interleukin-6 (IL-6) expression and decreased astrocyte proliferation by 30%. Additionally, miR-146a enhanced neuronal differentiation in co-cultured human neural stem cells, with miR-147b exhibiting stronger effects. Overall, miR-147b and miR-146a have been identified as key regulators of IL-1β-induced inflammatory responses in fetal astrocytes and epileptogenic brain tissue. Given miR-147b’s role in dampening inflammation and limiting astrocyte proliferation, it may hold therapeutic potential for inflammatory neurological disorders like temporal lobe epilepsy with hippocampal sclerosis (TLE-HS) and TSC.

Adding to the evidence for miRNA involvement in TSC brain pathology, members of the miR-34 family were significantly overexpressed in cortical tubers [48]. High-throughput RNA sequencing, protein-coding, and small non-coding RNAs were investigated in resected tubers from 12 TSC patients compared to 10 control brain samples. Widespread dysregulation of coding and ncRNAs was revealed, including 438 differentially expressed genes and 991 altered small non-coding RNAs. A significant overexpression of the miR-34 family—miR-34a, miR-34b, and miR-34c—was found among small RNAs. Immune-related genes, including those in the complement system and triggering receptor expressed on myeloid cells 1 (*TREM1*) signaling pathway, were upregulated, while genes linked to neurogenesis and glutamate receptor signaling were downregulated. From these miRNAs, miR-34b, in particular, has been shown to regulate neurite outgrowth in hippocampal neurons and trigger an inflammatory response in astrocytes, leading to significant alterations in TSC brain tissue. Therefore, it was indicated that miRNAs, especially the miR-34 family, are involved in the neuroinflammatory and neurodevelopmental irregularities seen in TSC. Collectively, it is underscored that the dysregulation of the miR-34 family plays a role in influencing the altered cellular and molecular environment of TSC brain lesions.

Building on the miR-34 family’s relevance, the expression of miR-34a was analyzed by obtaining tissue samples from 37 cortical tubers and comparing them to 27 control brain tissues obtained at autopsy [49]. To further investigate the functional role of miR-34a, its overexpression was evaluated in mice at embryonic day 18 (E18) to assess its impact on corticogenesis. Additionally, primary astrocytes derived from TSC patients and human-derived neuroblastoma cells (DH-SY5Y) were employed to investigate the influence of miR-34a on mechanistic target of rapamycin complex 1 (mTORC1) signaling. MiR-34a has been shown to be significantly overexpressed in cortical tubers of TSC patients during infancy as well as in the fetal TSC cortex. In the embryonic mouse model, overexpression of miR-34a resulted in a reduction in the migration of cells toward the cortical plate, suggesting an impairment in corticogenesis. Lastly, in TSC-derived human astrocytes, elevated miR-34a expression suppressed mTORC1 activity and led to a downregulation of key target genes, including ras proto-oncogene (*Ras*) and notch homolog 1 (*Notch1*). To conclude, it is revealed that miR-34a is overexpressed in cortical tubers during early brain development, negatively modulating mTORC1 activity and potentially disrupting corticogenesis in TSC.

Expanding on the understanding of *Tsc*’s role in miRNA processing, the epigenetic impact of *Tsc2* loss on miRNA processing in TSC was investigated using genetically modified *Tsc2*-deficient mouse embryonic fibroblasts (MEFs) [50]. Small RNA sequencing (smRNA-seq) was carried out to examine 752 miRNAs in two types of cells, namely *Tsc2*-expressing (wild-type) and *Tsc2*-deficient (knockout) MEFs. The results revealed that *Tsc2* deficiency caused a 5-fold boost in microprocessor activity, which converts pri-miRNAs to pre-miRNAs. Moreover, 37 miRNAs were increased, which was consistent with increased microprocessor activity. This effect was found to be reversed by GSK3β inhibitors, as well as mTORC1 and mTORC2 inhibitors (Rapamycin and Torin 1), implicating GSK3β as a downstream effector. *Tsc2*-deficient cells showed increased GSK3β levels, and mTOR inhibition led to higher GSK3β phosphorylation at Ser9, decreasing its activity. Lastly, the loss of *Tsc2* leads to widespread miRNA upregulation through mTORC1-driven GSK3β modulation of the microprocessor complex, with broad implications for cell growth, tumorigenesis, and cellular homeostasis in TSC.

Interestingly, the discovery of a new *TSC2* mutation and its subsequent impact on miRNA expression highlight the crucial role of miRNAs such as miR-199b-3p in modulating the mTOR pathway and driving TSC pathogenesis [51]. Skin tissue samples were obtained from a TSC patient with the variation c.1113delG in *TSC2* and from two healthy controls. In addition to the previously described c.2509_2512del mutation in *TSC1* from earlier studies, a novel de novo germline mutation in TSC2 (c.1113delG) was identified. Through qPCR, Western blotting, and functional assays, the study showed that mutations in *TSC1* and *TSC2* disrupt the *TSC complex*, leading to reduced expression of miR-199b-3p. The downregulation of miR-199b-3p was linked to increased mTOR expression and escalated activation of mTORC1 and mTORC2 signaling pathways. Restoring miR-199b-3p suppressed mTOR expression and decreased mTORC1/2 activity, highlighting its potential regulatory role. Therefore, mutations in *TSC1* and *TSC2* can lead to disease pathogenesis through epigenetic alterations, such as the reduced levels of miR-199b-3p, which lead to miRNA-mediated disruption of the mTOR pathway. 

The effects of *TSC1* and *TSC2* deficiency on miRNA expression were explored to investigate the epigenetic mechanisms involved in TSC pathogenesis that extend beyond the traditional mTOR signaling pathway [52]. Researchers identified 19 miRNAs with altered expression in *TSC1*-deficient cells, five in *TSC2*-deficient cells, and seven in control cells, with changes observed before and after treatment with rapamycin. Although miRNA levels changed, no corresponding alterations in mRNA expression were detected for four common target genes. This indicates that the dysregulation of miRNAs may influence gene regulation through post-transcriptional mechanisms independent of mTOR signaling. In addition, miR-21 has been identified as a pivotal contributor to tumor development in TSC [53]. Using Tsc2-deficient MEFs, human angiomyolipoma-derived 621-101 cells, and xenograft mouse models, it was shown that miR-21 expression was markedly increased in *TSC2*-deficient cells and was further elevated following rapamycin treatment, despite rapamycin’s role as an mTORC1 inhibitor. Functional inhibition of miR-21 using lentiviral knockdown (ZIP-21) and locked nucleic acid (LNA) antagomiRs reduced colony formation, increased sensitivity to apoptosis, and decreased tumor growth by over 50%. Co-treatment with rapamycin synergistically delayed tumor regrowth. Moreover, it was shown that miR-21 enhances mitochondrial function by modulating genes associated with oxidative phosphorylation and mitochondrial integrity, thereby supporting the survival of *TSC*-deficient tumor cells.

Lastly, a mouse model study focused on exploring the epigenetic regulation of mTOR signaling in TSC-associated renal cystogenesis [54]. Using the *Tsc2* conditional knockout mouse model (*Tsc2*^f/f; ksp-Cre) and *Tsc2*^−/−^ cell lines, it was investigated how miR-142-3p controls the expression of proline-rich AKT substrate of 40 kDa (PRAS40), a critical component of the mTOR complex. *Tsc2* loss was found to decrease miR-142-3p levels, causing elevated PRAS40 expression and enhanced renal cyst formation. Importantly, a DNA methylation inhibitor, decitabine, restored miR-142-3p, reduced PRAS40 expression, and inhibited cyst development at both molecular and histological levels. These findings indicate that in *Tsc2*-deficient conditions, miR-142-3p is epigenetically silenced via DNA methylation, leading to increased PRAS40 expression and mTOR hyperactivation. Restoration of miR-142-3p or inhibition of PRAS40 reduced cystogenesis. To conclude, a previously unrecognized epigenetic mechanism involved in TSC-associated kidney disease was revealed, and miR-142-3p was identified as a potential therapeutic target for inhibiting renal cyst development in TSC patients. The effects of microRNAs implicated in the pathogenesis of TSC are summarized in Table 1.

### 2.3. Epigenetic Alterations and Their Role in TSC Therapy

Emerging evidence suggests that epigenetic mechanisms, including DNA methylation and miRNAs, may offer therapeutic potential in TSC by modulating critical pathological pathways such as inflammation, tumorigenesis, and mTOR signaling. Rosuvastatin has shown potential in reversing *TSC2* promoter methylation. The therapeutic efficiency of rosuvastatin in reversing *TSC2* promoter methylation was assessed in TSC renal AML cells with different mechanisms of *TSC2* loss [34]. Two distinct cell types were analyzed: *TSC2*^−/−^ cells, which entirely lack tuberin due to gene deletion, and *TSC2*−/meth cells, where tuberin loss is caused by epigenetic silencing through promoter methylation. Rosuvastatin treatment in *TSC2*−/meth cells effectively induced demethylation of the *TSC2* promoter, restored tuberin expression, and reduced ras homolog family member A (RhoA) signaling, resulting in decreased cell proliferation. In contrast, in *TSC2*^−/−^ cells, rosuvastatin alone had no effect, and only when combined with the mTOR inhibitor rapamycin did it significantly reduce cell proliferation. These findings suggest that rosuvastatin can reverse epigenetic silencing in *TSC2*−/meth cells, while rapamycin contributes its anti-proliferative effect in cells with complete *TSC2* loss, pointing to a potential combinatorial therapeutic strategy in TSC-related tumors.

Intriguingly, the epigenetic impacts of everolimus, an mTOR inhibitor, on miRNA expression in TSC patients were assessed in SEGAs [55]. From 10 patients, serum samples were collected before and after three months of everolimus treatment. Also, miRNA profiles were analyzed using qPCR with TaqMan miRNA arrays to measure changes in expression levels. DNA was extracted from blood samples and subjected to sequencing to analyze mutations in *TSC1* and *TSC2* genes, followed by validation through Sanger sequencing. TSC was demonstrated as a disease characterized by a significantly changed serum miRNA profile. In addition, treatment with everolimus seemed to lead to *TSC*-specific alterations in miRNA expression levels, suggesting a distinct regulation of miR-222 and its involvement in modifying the phenotypes of TSC1 and TSC2. Interestingly, miR-222, known to regulate the mTOR pathway, exhibited varying expression depending on the underlying *TSC1* or *TSC2* mutation, with increased miR-222 expression associated with the milder clinical presentation in patients harboring *TSC1* mutations. Moreover, miR-142, which has been shown to inhibit the mTOR pathway, displayed increased levels alongside miR-130a and miR-130b after treatment with everolimus. These findings point to a role for miRNA dysregulation in TSC and associate specific miRNA profiles with treatment response.

Similarly, *TSC2*-deficient cells were examined to investigate the impact of rapamycin on miRNA-mediated pathways. MiRNA regulation was assessed in *TSC2*-deficient LAM cells during mTOR inhibition [56]. Rapamycin significantly increased miR-21 by enhancing primary miRNA-21 (pri-miR-21) to precursor microRNA-21 (pre-miR-21) conversion, a post-transcriptional process. This pro-survival effect, identified across different cell types, involves *TSC2*-deficient fibroblasts and is considered mTOR-dependent. Overall, rapamycin increased the levels of miR-21, a known pro-survival miRNA, thereby promoting cell survival in LAM and TSC. Thus, the induction of miRNAs may contribute to the therapeutic response of LAM and TSC patients to rapamycin treatment. In addition, the impact of sirolimus treatment on the serum miRNA profile in patients with TSC was examined, and the results were compared with those previously treated with everolimus [57]. The most significant changes in expression were the upregulation for miR-136-5p, miR-376a-3p, and miR-150-5p, which are linked to the RAS and MAPK signaling pathways. Remarkably, miR-136-5p and miR-150-5p appear to be linked to the mTOR pathway, especially its downstream effects linked to central nervous system pathology. However, further studies are required to clarify the relationship between upregulated miRNAs and treatment efficacy.

## 3. Epigenetic Mechanisms Underlying VHL

### 3.1. Overview of VHL

VHL disease is a multisystem neoplasia syndrome caused by germline mutations of the *VHL* gene in the short arm of chromosome 3 [20,58,59]. It is an autosomal dominant disorder, affecting 1 in 36,000 to 1 in 45,000 individuals [58]. The responsible mutated gene leads to VHL protein deficiency, which predisposes to tumor formation in various organs, namely the kidneys, the adrenal glands, the pancreas, the CNS and reproductive organs [20,58,59]. Nonetheless, according to the two-hit hypothesis, a somatic mutation or epigenetic transformation of the normal wild-type *VHL* allele is required for tumorigenesis [60]. Among the most commonly observed tumors in patients with VHL disease are CNS and retinal hemangioblastomas, endolymphatic sac tumors, pheochromocytomas, pancreatic neuroendocrine tumors and clear renal cell carcinomas (cRCCs), which are the most common type of inherited renal cancer [20,58,59,61].

The diagnosis of the syndrome is based on clinical criteria and can be confirmed by genetic testing [61,62,63]. In patients with a first-degree relative with documented VHL disease, a diagnosis can be made when there is at least one of the following clinical manifestations: RCC, pheochromocytoma, pancreatic neuroendocrine tumor, endolymphatic sac tumor, or retinal or CNS hemangioblastoma. However, in the case of a clear family history, a diagnosis is made if the patient presents with at least two clinical manifestations, one of which must be a hemangioblastoma [61]. Genetic detection of the mutated *VHL* variants can be implemented in patients with suspected VHL predisposition as a screening strategy, confirming the diagnosis regardless of the clinical findings [61,62,63]. Surveillance is of utmost importance in managing VHL disease and includes regular retinal and neurological examinations, combined with imaging and biochemical tests, namely plasma metanephrine and normetanephrine levels. Finally, the treatment of choice for VHL-related tumors is surgical removal, accompanied by appropriate adjunct therapies, if necessary [61].

### 3.2. Epigenetic Determinants of VHL Pathogenesis

There is limited research on the epigenetic mechanisms and alterations in VHL disease. The main results focus on the hypermethylation status of the wild-type *VHL* allele and the methylation of other genes involved in the manifestations of this multisystem syndrome. A study investigated the somatic mutations of the wild-type *VHL* allele through molecular-genetic analysis on 53 samples of tumors and their corresponding peripheral blood samples from 33 VHL patients [64]. The study’s main goals were detecting loss of heterozygosity, somatic mutations and VHL wild-type allele hypermethylation. Regarding the epigenetic part of the analysis, in 6 out of 18 tumors without loss of heterozygosity, the *NotI* site within exon 1 of the wild-type *VHL* allele was hypermethylated. This epigenetic alteration leads to the inactivation of the normal allele, subsequently initiating cancer development.

*VHL* gene hypermethylation has gathered a lot of attention, as it could be a possible candidate to explain the two-hit hypothesis. However, research efforts have expanded the panel of investigated genes that could be epigenetically altered and thus contribute to the pathogenesis of VHL disease. For instance, in a molecular analysis, methylation-specific PCR of the genes *FLIP*, *TSP1*, *DcR1*, *DcR2*, *DR4*, *DR5*, *CASP8* and *HIC1* was implemented on 20 neuroblastomas, 23 VHL-associated pheochromocytomas and 16 sporadic pheochromocytomas [65]. The epigenetic analysis revealed consistent methylation of *HIC1* and *CASP8* in pheochromocytomas, which was significantly more frequent in VHL pheochromocytomas compared to sporadic cases, underlying the presence of differing pathophysiological mechanisms between the two types of tumors. Moreover, the methylation status of *CASP8* and *HIC1* was significantly correlated in both neuroblastomas and phaeochromocytomas, with *CASP8* methylation observed exclusively in tumors exhibiting *HIC1* methylation. The frequency of methylation for the rest of the genes varied among the different types of tumors. The methylation of *the TSP1* gene may play a pivotal role in the pathophysiology of these tumors since there is evidence that it promotes angiogenesis. *CASP8*, *FLIP*, *DCR1*, *DCR2*, *DR4* and *DR5* gene methylation, on the other hand, disrupt the cell’s apoptotic mechanism.

In an attempt to define the epigenetic profiling of VHL-related and VHL-unrelated tumors, a study implemented CpG island methylation analysis on 29 VHL disease-related cRCCs, 20 sporadic VHL wild-type cRCCs, 13 papillary renal cell carcinomas (pRCCs), 6 samples of normal kidney tissue and 24 kidney cancer cell lines [66]. The mean of methylated CpG islands per tumor significantly differed among carcinomas, with the papillary RCCs demonstrating the highest and the VHL disease-related cRCCs the lowest value. Further analysis revealed the exact differentially methylated genes across the three tumor types. When comparing cRCCs and pRCCs, 14 genes (*RASSF1*, *SERPINE1*, *HOXA11*, *HOXC6*, *JAK3*, *PDGFRB*, *MMP2*, *ITGB1*, *CREB1*, *MYOD1*, *GSTM2*, *TNFRSF10C*, *SMARCA3* and *COL1A1*) were more methylated in the pRCCs, while 1 gene (*CDH1*) was more methylated in the cRCCs. There were also differences between VHL disease-related cRCCs and sporadic RCCs since 11 genes (*RASSF1*, *TWIST1 PITX2*, *CDH13*, *HS3ST2*, *TAL1*, *WT1*, *MMP2*, *DCC*, *ICA1* and *TUSC3*) were more frequently methylated in sporadic tumors and 1 (*GABRB3*) was more frequently methylated in VHL tumors. Therefore, it is evident that unique CpG methylation profiling could be of utmost importance in unraveling tumorigenesis, screening, and diagnosis.

Even though there is evidence of epigenetic alterations in VHL disease that may contribute to clinical manifestations, certain analyses yield null results. In the context of the two-hit hypothesis validation, a study focused on the inactivation of the *VHL* wild-type allele in familial and sporadic cerebellar hemangioblastomas [67]. For this purpose, nine tissue samples of surgically resected cerebellar hemangioblastomas were analyzed. These samples were obtained from three patients with VHL disease and five patients with sporadic hemangioblastomas. DNA was extracted from each sample and subjected to multiple analyses, including single-strand conformation polymorphism, DNA sequencing, microsatellite polymorphism analysis, and DNA methylation analysis. No epigenetic alterations involving hypermethylation of the CpG islands in exon 1 of the VHL gene were detected. However, further analyses should investigate the null results due to the study’s inadequate tissue samples. Similarly, a recent comparative study utilizing genome-wide DNA methylation profiling on 11 sporadic and 11 VHL-disease-related endolymphatic sac tumors did not demonstrate any differences in the DNA methylation status [68]. This observation could indicate the presence of common epigenetic landscapes between the compared clinical entities. Moreover, VHL promoter methylation was only detected in sporadic cases.

Another study on CNS hemangioblastomas found no hypermethylation of the *VHL* wild-type allele promoter [69]. Researchers analyzed a larger cohort comprising 29 surgically resected hemangioblastomas associated with VHL disease and 13 sporadic hemangioblastomas, along with the corresponding peripheral blood samples. The aim was to identify potential germline mutations in the *VHL* gene. Genetic analysis included single-strand conformational polymorphism, loss of heterozygosity and methylation analyses. A possible interpretation of the null results could lie in the failure of amplification of the promoter fragment. In the same line, molecular analysis of the *VHL* gene from 57 surgically removed CNS capillary hemangioblastomas yielded no hypermethylation of the promoter due to the insufficient sample size (only two patients with VHL disease) and failure of amplification of the promoter fragment [70]. Finally, the same results were reproduced by a comparative study analyzing 11 VHL-related and 21 sporadic hemangioblastomas, where hypermethylation was detected only in sporadic cases [71]. The epigenetic mechanisms implicated in the pathogenesis of VHL disease are summarized in Table 2.

## 4. Mechanisms of Epigenetic Dysregulation in A-T

### 4.1. Overview of A-T

A-T is a rare type of phacomatosis, following the autosomal recessive pattern of inheritance [19,72,73]. The prevalence rate varies from 1 in 300,000 to 1 in 40,000 live births [19,74]. The phenotype of the disease is a result of pathogenic variants of the ataxia telangiectasia mutated (*ATM*) gene in chromosome 11q22-23, which encodes a serine/threonine kinase. This enzyme is involved in multiple processes in the cell cycle, such as DNA repair, chromatin remodeling and apoptosis. Thus, cells carrying mutations are prone to DNA damage by reactive oxygen species, toxins and ionizing radiation [19,72,73]. The patients present with various neurological defects, namely cerebellar ataxia, mild cognitive compromise, difficulty in swallowing and oculomotor apraxia, with the majority of affected individuals requiring wheelchair assistance by 10 years of age. Other clinical features include dermatologic manifestations, such as telangiectasias and café-au-lait macules. Affected individuals also commonly present with primary immunodeficiency, which predisposes them to recurrent pulmonary infections. Additionally, there is an increased risk for certain malignancies, particularly lymphomas, leukemias, and breast cancer [19,72,75,76].

Currently, the diagnostic methods include a combination of representative clinical and lab findings. In more detail, the presence of neurological symptoms, namely ataxia, defective eye movement control and postural instability, should be combined with one of the following: telangiectasia, intermittent respiratory infections or specific laboratory abnormalities; these include rising alpha-fetoprotein levels, decreased immunoglobulins, cerebellar atrophy in the MRI, spontaneous radiation-induced chromosomal aberrations and reduced survival of X-ray-exposed cultured lymphocytes and fibroblasts. Detection of *ATM* gene mutations or ATM protein deficiency confirms the diagnosis [19]. Regarding the management of the disease, the available interventions only offer palliative care. These include anti-Parkinson and anti-epileptic drugs, early and aggressive interventions regarding respiratory infections, gamma globulin therapy, regular immune system evaluations, proper vaccination and gastrostomy tube insertion [19,77,78,79,80].

### 4.2. Epigenetic Alterations in A-T: A Pathogenic Link

#### 4.2.1. DNA Methylation

DNA methylation has been investigated to some extent in the context of A-T since it could shape, along with other factors, the phenotype of the disorder. A study focused on 5-hydroxymethylcytosine (5hmC) levels in cerebellar neurons of A-T patients and *Atm*^−/−^ mice [79]. For this purpose, the 5hmC levels of Purkinje cells were compared among cerebellum samples from four pairs of individuals, one succumbing to a cause other than brain disease and the other to A-T. Individuals with A-T and *Atm*^−/−^ mice displayed a significant reduction of 5hmC compared to healthy controls and an overall shift in 5hmC-enriched regions. Consequently, genes involved in neuron-specific processes regarding differentiation and nervous system development were downregulated. Moreover, further analyses revealed that the enzyme TET1, which converts 5-methylcytosine to 5hmC, undergoes phosphorylation by the ATM protein in response to DNA damage by y-irradiation. As expected, the increase of 5hmC in ATM-deficient cells was significantly less than in control cells. Further experiments showed that knockdown of TET1 may be involved in neuronal death and cell cycle reentry, as well as that *ATM* deficiency leads to region-specific loss of 5hmC in the cerebellum, especially in Purkinje cells, altering epigenetic regulation of neuron-specific genes and reducing their expression. Thus, it is evident that TET1, an ATM-phosphorylated enzyme, participates in chromatin formation. Therefore, *ATM* deficiency leads to 5hmC reduction in Purkinje cells, which may mediate neurodegeneration in A-T.

Another molecular analysis aimed to underscore the differences in DNA methylation and gene expression among individuals with mild A-T, classic A-T and healthy controls [80]. The researchers recruited eight individuals with mild or classic A-T and four healthy participants, isolating peripheral blood mononuclear cells for genome-wide methylation analysis with modified reduced representation bisulfite sequencing. In individuals with A-T exhibiting the classic phenotype, gene expression analysis revealed distinct regulatory patterns. Genes involved in apoptosis, as well as those associated with phosphate and nitrogen metabolic processes, were downregulated. In contrast, genes related to immune system function were upregulated. Regarding genes that control lymphocyte development and B-cell function, those were downregulated among individuals with the A-T phenotype compared to healthy controls. In contrast, genes that participate in cell activity and influence the rate of response to stimulus were upregulated in A-T participants. Further investigations identified 20 differentially expressed genes in individuals with A-T compared to controls. These genes are implicated in key clinical manifestations of A-T, including ataxia, malignancy, and B cell dysfunction. All observed differences in gene expression were consistent with DNA methylation profiling, which revealed distinct patterns of CpG methylation among the phenotypes. Thus, results indicate that DNA methylation can play a pivotal role in gene expression and pathogenesis of A-T.

#### 4.2.2. Histone Modifications

Histone modifications, specifically histone deacetylation and methylation, are contributing factors to chromatin conformation in A-T (Table 3). For instance, there is evidence that histone methylation induces epigenetic alterations implicated in neurodegeneration in A-T. The implementation of immunostaining and Western blots in a molecular study revealed an increase in trimethylated histone 3 on Lys 27 in both human and mouse ATM-deficient cells compared to controls [81]. The mediator of this phenomenon is the enzyme PRC2 and its core catalytic component, EZH2. In normal cells, ATM phosphorylates EZH2, leading to PRC2 instability and its consequent inactivation. As predicted, the hypermethylation of various gene promoters led to gene downregulation in ATM-deficient cells, as shown by RNA microarray analysis. Moreover, the knockdown of EZH2 promoted neuronal survival and prevented cell cycle reentry. Yet, importantly, the overall shift in chromatin conformation in A-T has been correlated to the vulnerability of ATM-deficient cells to radiation-induced DNA lesions. For this purpose, two A-T lymphoblastoid lines and one wild-type line were exposed to X-rays to induce DNA damage [82]. Afterwards, cells were post-treated with trichostatin A, cytosine arabinoside, an inhibitor of DNA repair synthesis, or a combination of both substances. A portion of irradiated cells did not undergo post-treatment. Results showed that ATM-deficient cells were more sensitive to radiation and benefited from trichostatin A post-treatment, while cytosine arabinoside counteracted this effect. Thus, the induction of chromatin decondensation in ATM-deficient cells makes heterochromatin more accessible to DNA repair enzymes, proving that chromatin conformation status is a significant mediator of the pathogenesis of the disease.

In the same line with the aforementioned studies, another research investigated whether the nuclear accumulation of HDAC4 in ATM deficiency is associated with neurodegeneration in A-T [83]. This deacetylase has been shown to mediate brain development and promote neuronal survival. An integrative analysis was performed using cerebellar tissue sections from both humans (with and without ataxia–telangiectasia) and mice (*Atm*^+/+^ and *Atm*^−/−^). These sections underwent immunostaining using an antibody against HDAC4. Interestingly, ATM deficiency induced HDAC4 nuclear accumulation due to the enzyme’s hypophosphorylation, which reduced acetylation on histones H3 and H4 in promoter sites. This modification led to compact chromatin conformation, resulting in gene suppression and neurodegeneration. Specifically, the genes *CREB* and *MEF2A*, which encode prosurvival transcription factors, were found to be inactivated in the presence of nuclear HDAC4 in ATM-deficient cells. This was supported by immunoprecipitation and quantitative PCR analyses, which demonstrated reduced promoter occupancy by these transcription factors. Further analysis revealed that inhibition of HDAC4 with trichostatin A and the presence of this particular deacetylase in cytoplasm promoted cell survival. In conclusion, HDAC4 accumulation and increased PRC2 function in A-T create a compact chromatin conformation, suppressing genes that prevent neurodegeneration. Unraveling these epigenetic alterations may indicate specific molecular pathways for potential therapeutic interventions.

## 5. Conclusions

This comprehensive review underscores the critical role of epigenetic mechanisms in the pathogenesis, prognosis and management of neurocutaneous syndromes, with a focus on TSC, VHL, and A-T. Although well-characterized genetic mutations primarily drive these disorders, emerging evidence highlights that DNA methylation, histone modifications, and ncRNA regulation contribute significantly to the phenotypic variability and disease progression observed in affected individuals. In TSC, dysregulation of the mechanistic mTOR pathway and epigenetic modifications such as DNA methylation and miRNA alterations provide new insights into its molecular etiology. In VHL, epigenetic silencing of the VHL gene through promoter hypermethylation plays a key role in tumorigenesis. Furthermore, in A-T, reduced levels of 5hmC, combined with abnormal histone deacetylation and methylation, lead to deregulated gene expression. The marked phenotypic heterogeneity seen in these syndromes suggests that epigenetic dysregulation may underlie the incomplete genotype–phenotype correlations observed. Future research should integrate multi-omic and single-cell approaches to uncover cell-specific epigenetic signatures in neurocutaneous syndromes. Longitudinal studies are needed to identify epigenetic biomarkers for disease monitoring and assess whether such alterations influence therapeutic response. Targeting epigenetic modifiers may offer novel, personalized strategies, especially in treatment-resistant cases. Integrating epigenetic profiling with genetic data holds promise for uncovering novel therapeutic targets, thereby enabling the development of more precise, individualized treatment strategies for these complex disorders.

## Figures and Tables

**Table 1:** Effects of microRNAs implicated in the pathogenesis of tuberous sclerosis complex.

MicroRNAs	Level	TSC Phenotype	Regulators	Biological Role	Ref.
miR-23a; miR-34a	↑	Epilepsy	TSC1	Directly target the 3′ UTR of the TSC1-suppressing hamartin expression that increases seizure susceptibility in cortical tubers	[41]
miR-142-3p; miR-142-5p; miR-223-3p; miR-200b-3p; miR-32-5p	↑	Epileptogenic OH tubers	SLC12A5; SYT1; GRIN2A; GRIN2B; KCNB1; SCN2A; TSC1; MEF2C	Downregulate SLC12A5, SYT1, GRIN2A, GRIN2B, KCNB1, SCN2A, TSC1 and MEF2C that are implicated in epilepsy risk	[42]
miR-320d	↓	SEGAs	MMP2	Increase MMP2 expression in human fetal astrocytes	[43]
miR146a; miR147b	↑	Epilepsy	TIMP2; TIMP3; TIMP4; MMP3	Increased mRNA expression of TIMP2, TIMP3, and TIMP4, along with decreased expression of MMP3, contributes to the restoration of blood–brain barrier integrity and extracellular matrix homeostasis	[44]
miR-9-5p; miR-124-3p; miR-132-3p	↑	Renal angiomyolipoma	BCL2L11	BCL2L11 downregulation leads to AML pathogenesis	[45]
miR-34a; miR-34b; miR-34c	↑	Cortical tubers	Neurogenesis; glutamate receptor signaling	The miR-34 family may regulate neurogenesis and glutamatergic signaling in TSC; their dysregulation may contribute to common TSC comorbidities	[48]
miR-34a	↑	Cortical tubers	RAS; NOTCH1; mTORC1	mTORC1 suppression along with downregulation of RAS and NOTCH1 signaling pathways disrupts corticogenesis in TSC	[49]
miR-199b-3p	↓	Renal angiomyolipoma; cardiac rhabdomyoma	mTORC1; mTORC2	Hyperactivation of both mTORC1 and mTORC2 causes excessive proliferation and resistance to apoptosis	[51]
miR-21	↑	Hamartomas; TSC-related tumors	mTORC1; mitochondria	In vitro inhibition of miR-21 reduced colony formation, increased apoptosis sensitivity, and decreased tumor growth	[53]
miR-142-3p	↓	Renal cystogenesis	PRAS40	Increased PRAS40 expression and mTOR hyperactivation; cystogenesis promotion	[54]
miR-146a; miR-147b; miR-155	↑	Astrocyte-mediated inflammatory response	COX-2; IL-6	miR155 showed pro-inflammatory effects, while miR146a showed anti-inflammatory effects. miR146a and miR-147b lead to a reduction in COX-2 and IL-6 expression and decreased astrocyte proliferation by 30%	[46,47]

miRNA: microRNA, TSC1: tuberous sclerosis 1, 3 UTR: 3′ untranslated region, OH—Onset/AMT-hot: seizure onset zones that show increased AMT uptake on PET scans, SLC12A5: solute carrier family 12 member 5, SYT1: synaptotagmin 1, GRIN2A: glutamate ionotropic receptor NMDA type subunit 2A, GRIN2B: glutamate ionotropic receptor NMDA type subunit 2B, KCNB1: potassium voltage-gated channel subfamily B member 1, SCN2A: subependymal giant cell astrocytomas, MEF2C: myocyte enhancer factor 2C, SEGAs: subependymal giant cell astrocytomas, MMP2: matrix metalloproteinase, TIMP2: tissue inhibitors of metalloproteinases, BCL2L11: BCL-2-interacting mediator of cell death, AML: angiomyolipoma, RAS: ras proto-oncogene, NOTCH1: notch homolog 1, mTORC1: mechanistic target of rapamycin complex 1, mTORC2: mechanistic target of rapamycin complex 2, PRAS40: proline-rich AKT substrate of 40 kDa, COX-2: cyclooxygenase-2, IL-6: interleukin-6.

**Table 2:** Epigenetic mechanisms implicated in the pathogenesis of von Hippel–Lindau.

Epigenetic Mechanism	Key Mediator	Impact	Effect of Modification	Ref.
DNA methylation	Methylation of the *NotI* site within exon 1 of the wild-type *VHL* allele	Yes	Wild-type *VHL* allele inactivation leads to RCC, hemangioblastoma, pheochromocytoma and pancreatic tumor development	[64]
DNA methylation	Methylation of *FLIP*, *TSP1*, *DcR1*, *DcR2*, *DR4*, *DR5*, *CASP8 and HIC1* promoters	Yes	Methylation of genes’ promoters alters their expression, promoting angiogenesis and causing apoptosis dysregulation, leading to pheochromocytoma development	[65]
DNA methylation	Promoter CpG islands methylation of multiple tumor suppressor genes	Yes	Unique CpG methylation patterns indicate differences in gene inactivation and tumorigenesis between VHL-related and VHL-unrelated RCCs	[66]
DNA methylation	Methylation of CpG islands of exon 1 of the *VHL* wild-type allele	No	Not applicable	[67]
DNA methylation	Methylation of *VHL* wild-type allele promoter	No	Not applicable	[69]
DNA methylation	Methylation of *VHL* wild-type allele promoter	No	Not applicable	[70]
DNA methylation	Methylation of *VHL* wild-type allele promoter	No	Not applicable	[71]
DNA methylation	Whole genome methylation status and methylation of *VHL* wild-type allele promoter	No	Not applicable	[68]

CpG: cytosine-phosphate-guanine; DNA: deoxyribonucleic acid; RCC: renal cell carcinoma; VHL: von Hippel–Lindau.

**Table 3:** Epigenetic mechanisms implicated in the pathogenesis of ataxia–telangiectasia.

Epigenetic Mechanism	Key Mediator	Impact	Effect of Modification	Ref.
DNA methylation	5hmC levels and 5hmC-enriched DNA regions	Yes	Reduced levels of 5hmC and alteration of 5hmC patterns in Purkinje cells lead to neurodegenration	[79]
DNA methylation	Methylation of promoters’ CpG islands of genes involved in immune responses, growth and apoptotic pathways	Yes	Altered methylation of genes involved in immune responses, growth and apoptotic pathways regulates their expression and leads to apoptosis dysregulation, immune system modifications, immunodeficiency, ataxia and malignancy (particularly B-cell lymphomas)	[80]
Histone modification	EZH2-mediated H3K27 trimethylation at multiple neurotrophic genes’ promoters	Yes	Downregulation of neurotrophic genes leads to neurodegeneration	[81]
Histone modification	HDAC4-mediated deacetylation of histone 3 and 4 at promoters of genes *Mef2A* and *Creb*	Yes	Downregulation of the pro-survival transcription factors CREB and MEF2A contributes to neurodegeneration	[83]
Histone modification	Overall chromatin conformation of the whole genome	Yes	ATM-deficient cells exhibit increased susceptibility to radiation-induced DNA lesions, which are often sequestered in regions less accessible to DNA repair machinery, thereby contributing to an elevated risk of malignancy	[82]

5hmC: 5′-hydroxymethylcytosine; CpG: cytosine-phosphate-guanine; CREB: cAMP response element-binding protein; DNA: deoxyribonucleic acid; HDAC4: histone deacetylase 4; EZH2: enhancer of zest homolog 2; H3K27: histone H3 protein-lysine 27; MEF2A: myocyte enhancer factor 2A.

## Data Availability

Not applicable.

## Abbreviations

The following abbreviations are used in this manuscript:

3 UTR	3′ untranslated region
5hmC	5′-hydroxymethylcytosine
A-T	ataxia–telangiectasia
AML	angiomyolipoma
AMT PET	a-methyltryptophan positron emission tomography
ATM	ataxia telangiectasia mutated
BCL2L11	BCL-2-interacting mediator of cell death
CD44v6	variant isoform of the CD44 cell surface glycoprotein
CNS	central nervous system
COX-2	cyclooxygenase-2
CpG	cytosine-phosphate-guanine
cRCCs	clear renal cell carcinomas
CREB	cAMP response element-binding protein
DNA	deoxyribonucleic acid
EGFR	Epidermal Growth Factor Receptor
EZH2	enhancer of zest homolog 2
GRIN2A	glutamate ionotropic receptor NMDA type subunit 2A
GRIN2B	glutamate ionotropic receptor NMDA type subunit 2B
GSK3β	glycogen synthase kinase 3 beta
HDAC	histone deacetylase
HM450	HumanMethylation450
HMB45	human melanoma black 45
IL-1β	interleukin-1b
IL-6	interleukin-6
KCNB1	potassium voltage-gated channel subfamily B member 1
LAM	lymphangioleiomyomatosis
LNA	locked nucleic acid
Lys	lysine
MAPK	mitogen-activated protein kinase
MEFs	mouse embryonic fibroblasts
MEF2A	myocyte enhancer factor 2A
MEF2C	myocyte enhancer factor 2C
miRNA	microRNA
MMP	Matrix Metalloproteinase
mTOR	mechanistic target of rapamycin
mTORC1	mechanistic target of rapamycin complex 1.
NC—Non-onset/AMT-cold	brain regions that are not seizure onset zones and show reduced AMT uptake on PET imaging
ncRNA	non-coding RNA
NF1	Neurofibromatosis type 1
NF2	Neurofibromatosis type 2
non-CpG	non-cytosine-phosphate-Guanine
NOTCH1	notch homolog 1
OC—Onset/AMT-cold	seizure onset zones that also show reduced AMT uptake on AMT PET scans
OH—Onset/AMT-hot	seizure onset zones that show increased AMT uptake on PET scans
PCR	Polymerase Chain Reaction
PRC2	Polycomb Repressive Complex 2
PRAS40	proline-rich AKT substrate of 40 kDa
pRCCs	papillary renal cell carcinomas
pre-miR-21	precursor microRNA-21
pri-miR-21	primary microRNA-21
qPCR	quantitative Polymerase Chain Reaction
RAS	ras proto-oncogene
RCC	renal cell carcinoma
RhoA	ras homolog family member A.
RNA	ribonucleic acid
RNAseq	RNA sequencing
RT-PCR	Reverse Transcription Polymerase Chain Reaction
SCN2A	Sodium Channel Subunit Alpha 2
SEGAs	subependymal giant cell astrocytomas
SENs	subependymal nodules
SLC12A5	solute carrier family 12 member 5
smRNA-seq	small RNA sequencing
SNP	single nucleotide polymorphism arrays
SYT1	Synaptotagmin 1
TIMPs	tissue inhibitors of metalloproteinases
TLE-HS	temporal lobe epilepsy with hippocampal sclerosis
TREM1	triggering receptor expressed on myeloid cells 1
TSC	tuberous sclerosis complex
TSC2-/meth	TSC2 gene that is silenced by promoter methylation
TSC2^+/−^	heterozygous mutations in the TSC2 gene
TSC2^+/+^	tuberous sclerosis complex 2 wild-type homozygous genotype
VEGF-D	vascular endothelial growth factor-D.
VHL	von Hippel–Lindau
WES	whole-exome sequencing
ZIP-21	Zinc-finger Interfering Plasmid targeting miR-21
